# Synapsid tracks with skin impressions illuminate the terrestrial tetrapod diversity in the earliest Permian of equatorial Pangea

**DOI:** 10.1038/s41598-023-27939-z

**Published:** 2023-01-20

**Authors:** Gabriela Calábková, Jakub Březina, Vojtěch Nosek, Daniel Madzia

**Affiliations:** 1 Department of Geology and Paleontology, Moravian Museum, Zelný Trh 6, 659 37 Brno, Czech Republic; 2 Department of Geological Sciences, Faculty of Science, Masaryk University, Kotlářská 267/2, 611 37 Brno, Czech Republic; 3 Department of Archaeology and Museology, Faculty of Arts, Masaryk University, Joštova 220/13, 662 43 Brno, Czech Republic; 4 Institute of Paleobiology, Polish Academy of Sciences, Twarda 51/55, 00-818 Warsaw, Poland

**Keywords:** Palaeontology, Taxonomy, Biodiversity

## Abstract

Lower Permian deposits of the Boskovice Basin in the Czech Republic have long been renowned for extraordinarily abundant specimens of discosauriscid seymouriamorphs, some of which showing exceptional preservation, including widespread soft tissues. The only other tetrapods from the strata are represented by rare temnospondyls. However, recent fieldwork in the Asselian (lowermost Permian) of the Boskovice Basin has yielded a diverse assemblage of tetrapod tracks, illuminating a hidden terrestrial tetrapod diversity. Here, we describe well-preserved isolated tracks, manus-pes couples, and a slab with trackways composed of approximately 20 tracks in at least four different directions belonging to early-diverging, or ‘pelycosaur-grade’, synapsids. The material originates from three localities situated within the Letovice and Padochov formations and is assignable to the ichnotaxon *Dimetropus*. The best-preserved specimen further shows rare skin impressions, which have not been observed from the hands or feet of early-diverging mammal-line amniotes before. The new material adds to the scarce record of synapsids from the Carboniferous/Permian transitional interval of equatorial Pangea. At the same time, it highlights the significance of the ichnological record of the Boskovice Basin which has long been neglected despite offering evidence for the presence of diverse faunal components that have not been reported from these basinal deposits before.

## Introduction

The Asselian (lowermost Permian) strata of the Boskovice Basin in the Czech Republic are recognized for their fossil-rich assemblages of discosauriscid seymouriamorphs some of which exhibit exceptional preservation, including soft tissues, such as external gills and eye structures^1–4^. Discosauriscids have been unearthed from several horizons within the basin (e.g., Ref.^5^, Fig. 2). They are represented by four or five species: *Discosauriscus austriacus*^6^, *Discosauriscus pulcherrimus*^7^, *Makowskia laticephala*^8^, *Spinarerpeton brevicephalum*^9^, and a possible new species from Kochov-Horka in the Letovice Formation^10^, though the abundance of these taxa in the basinal strata is extremely uneven. For example, *M. laticephala*, *S. brevicephalum*, and the potential new taxon from Kochov-Horka are each based on a single specimen, whereas *Discosauriscus austriacus*, the most abundant tetrapod taxon in the Boskovice Basin, is known from hundreds of individuals, including larvae, juveniles, and a specimen interpreted as an early adult^1,3,9^. Tetrapods other than discosauriscids are extremely rare in the Boskovice Basin, and are evidenced only by a few specimens of temnospondyls, including several poorly preserved ‘branchiosaurs’^11^, a zatracheid cf. *Dasyceps*^12^, and the stereospondylomorph *Sclerocephalus stambergi*^13^.

All these specimens have been unearthed from grey lacustrine horizons (see Refs.^14,15^). The terrestrial faunal components of Permian age of the Boskovice Basin are essentially unknown. However, recent fieldwork conducted in the Asselian strata at various localities situated within the basin has yielded diverse assemblage of tetrapod tracks and trackways that have been made by terrestrial tetrapods^5,16,17^.

Here we report a new assemblage of tracks of varying degrees of preservation originating from three localities of the Letovice and Padochov formations, which can be attributed to the ichnotaxon *Dimetropus*. The specimens provide the first evidence of synapsids in the lowermost Permian of the Boskovice Basin. From a general perspective, early-diverging, or ‘pelycosaur-grade’, synapsids are extremely rare in the Permo-Carboniferous basins of the Czech Republic, and all originate from Pennsylvanian (upper Carboniferous) coal seams^18–22^. Among these, the ophiacodontid *Archaeothyris* sp. from the Moscovian (Westphalian D) of Nýřany (Pilsen Basin, Kladno Formation)^19,23,24^, the stratigraphically oldest European sphenacodontid *Macromerion schwarzenbergii*^18,25^ and the historically oldest European edaphosaurid *Bohemiclavulus mirabilis*^20,26^, both from the Gzhelian (Stephanian B) of Kounov (Kladno-Rakovník Basin, Slaný Formation), and the largest known edaphosaurid, referred to as ‘*Ramodendron obvispinosum*’, from the Gzhelian (Stephanian C) of Oslavany (Boskovice Basin, Rosice-Oslavany Formation)^21,26^, likely represent the most significant specimens.

In this contribution we provide the full description of the synapsid tracks and trackways, illustrate the material using three-dimensional modeling, and assess it especially with respect to the taxonomic affinities of their trackmakers.

*Institutional Abbreviations* MZM, Moravian Museum, Brno, Czech Republic.

### Geological setting

**Figure 1:**
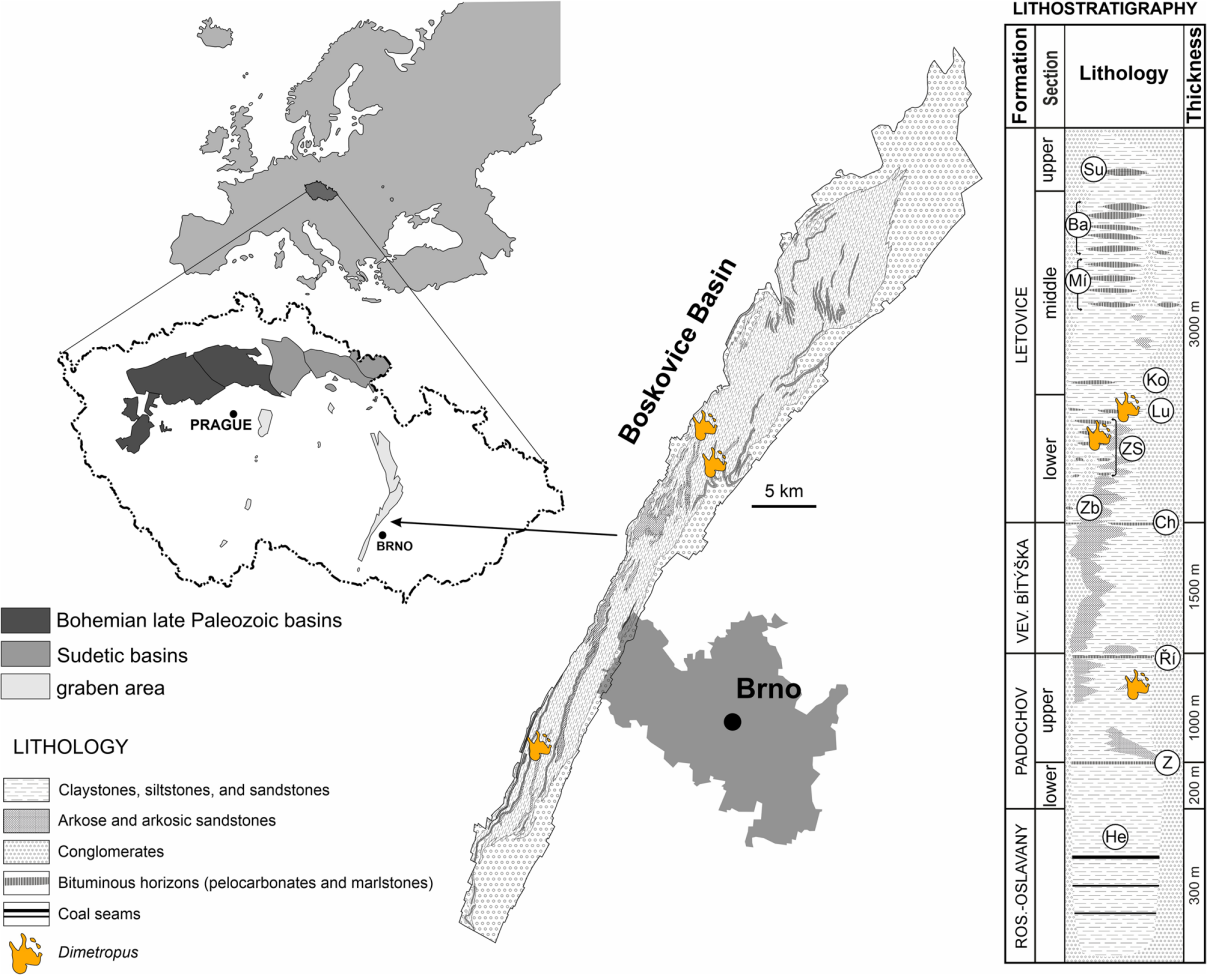
Location of the studied area and the distribution of *Dimetropus* tracks in the Boskovice Basin. Horizons: *He* Helmhacker, *Z* Zbýšov, *Ří* Říčany, *Ch* Chudčice, *Zb* Zbraslavec, *ZS* Zboněk-Svitávka, *Lu* Lubě, *Ko* Kochov, *Mí* Míchov, *Ba* Bačov, *Su* Sudice. Modified after^5^. The geological map and lithostratigraphy follow^14,28,29^. Chronostratigraphy follows^45^ and Jirásek (pers. comm.).

The Boskovice Basin represents a NNE–SSW-oriented depression situated in the eastern margin of the Bohemian Massif that is about 100 km long and 3–10 km wide (Fig. 1). Sedimentation started in the southern part of the basin during the Gzhelian (latest Carboniferous) and continued uninterrupted towards to north through the Asselian (earliest Permian)^27–29^. The marginal facies are composed of the Balinka conglomerates (partly including breccias) in the west and the Rokytná conglomerates (also partly composed of breccias) in the east^28,29^ that are interpreted as an alluvial fan system that prograded towards the basin, diachronously with the sedimentation of all formations (e.g. Refs.^30,31^).

The intrabasinal sedimentary complex is composed of cyclically arranged fluvial to fluviolacustrine clastic deposits. They are mostly red-colored, with the co-occurrence of grey-colored units indicating short-term semi-humid oscillations^28,29,32^. The cumulative thickness of the deposits is estimated to have been up to 5–6 km thick^28–30^. However, seismic data indicate that the current maximum thickness for a single section of the basin is around 3 km^33,34^.

The synapsid footprints described here originate from the upper section of the Padochov Formation and the lower section of the Letovice Formation, which are Asselian (earliest Permian) in age (Fig. 1). The vegetation recorded from the Padochov and Letovice formations includes pteridosperm- and conifer-dominated assemblages^32,35^. Faunal components of the Padochov Formation consist of bivalves, clam shrimps (‘conchostracans’), insects, acanthodians, xenacanthids, branchiosaurids, and indeterminate, very rare finds of discosauriscids (e.g. Refs.^12,15,36–45^). The Letovice Formation material additionally comprises actinopterygians, discosauriscids, and several members of temnospondyls (e.g. Refs.^4,8,13,38,39,45–50^).

## Methods

The protocol for obtaining measurements and applied terminology follow^51^. The specimens were measured using a digital caliper and the software ImageJ. Only clearly defined parameters were measured on the tracks to prevent distortion of the resulting values (see Table 1).

**Table 1:** Ichnological parameters of *Dimetropus* tracks. *L* length, *m* manus, *p* pes, *psL* palm/sole length, *psW* palm/sole width, *W* width, *γ I–V* interdigital angle I–V (°), *I–V L* length of digits I–V. Measurements in mm and degrees.

Tracks parameters		L	W	I L	II L	III L	IV L	V L	psL	psW	γ I–V
MZM Ge 29610	m	103.2	102.8	23.4	26.5	33.7	36.6	17.7?	69.9	84.1	54.9°
	p	124.3	104.2	20.9?	27.2	32.3	42.6	31	88.4	86.1	56.2°
MZM Ge 33071	?	–	94	28	26.8	36.8	46.5	–	–	–	–
MZM Ge 33072	m	90.5	97.6	–	20.8	–	–	–	71.9	94.7	78.5°
	p	112.3	88.9	–	23	29.7	38.2	–	85.2	85	–
MZM Ge 33073	?	149.9	82.7	–	–	–	–	–	98	82.7	–
MZM Ge 33074	track B	138	115	18.5	22.3	28.4	29.6	24.5	111.1	93.1	64.4°
	track C	124.1	86.8?	19.2	–	24.3	33	–	93.3	85.4	–

The protocol for three-dimensional (3D) modeling of the samples follows^52^. First, we took 72 photographs of MZM Ge 29610, MZM Ge 33071, MZM Ge 33072, and MZM Ge 33073 using a full frame camera Nikon D750 (lens Tamron 24–75 mm, F2.8). Each photograph was taken in two elevation positions to fully cover the surface of the samples. Obtained images were then used for reconstruction of 3D photogrammetric models through Agisoft Metashape PRO 1.7.1. This procedure was complemented with scans using the geo-referenced marker grid matrix, which provides a greater precision (and automation of the reconstruction process) than the usual geo-referencing of the model through one or more scale bars. All models have been reconstructed in the highest possible quality to the final resolution of 3.5 million polygons. Resulting 3D models were visualized and interpreted using CloudCompare 2.10.

Since the concentric scanning technique of SfM data acquisition (more on SfM in e.g. Ref.^53^) used for the rest of the samples could not be applied due to the location of the concave epirelief of the specimen MZM Ge 33074 in the field, we used an acquisition method with a distribution of image positions similar to that of ranging prospection used for larger objects (e.g. Ref.^54^). A total of 140 photos were taken in an axis as perpendicular to the plate surface as possible, with sufficient overlap using a Canon PowerShot G7 X Mark II camera and the resolution of 20.1 Mpx. These images subsequently went through the same reconstruction process as in the case of the other samples, with an emphasis on the quality of the resulting scan, not on the speed of calculation, and were evaluated and visualized in a similar way. Further, the specimen MZM Ge 33074 was visualized through the PCV algorithm (+ Z hemisphere, 256 rays, render context resolution 1024).

Three-dimensional models (in the form of meshes) obtained from physical samples using the Structure from Motion method were uploaded to the MorphoSource data archive: https://www.morphosource.org/projects/000489812/about?locale=en.

## Systematic paleontology

Amniota^55^.

Synapsida^56^.

*Dimetropus*^57^.

*Dimetropus leisnerianus*^58^.

### Referred material

**Figure 2:**
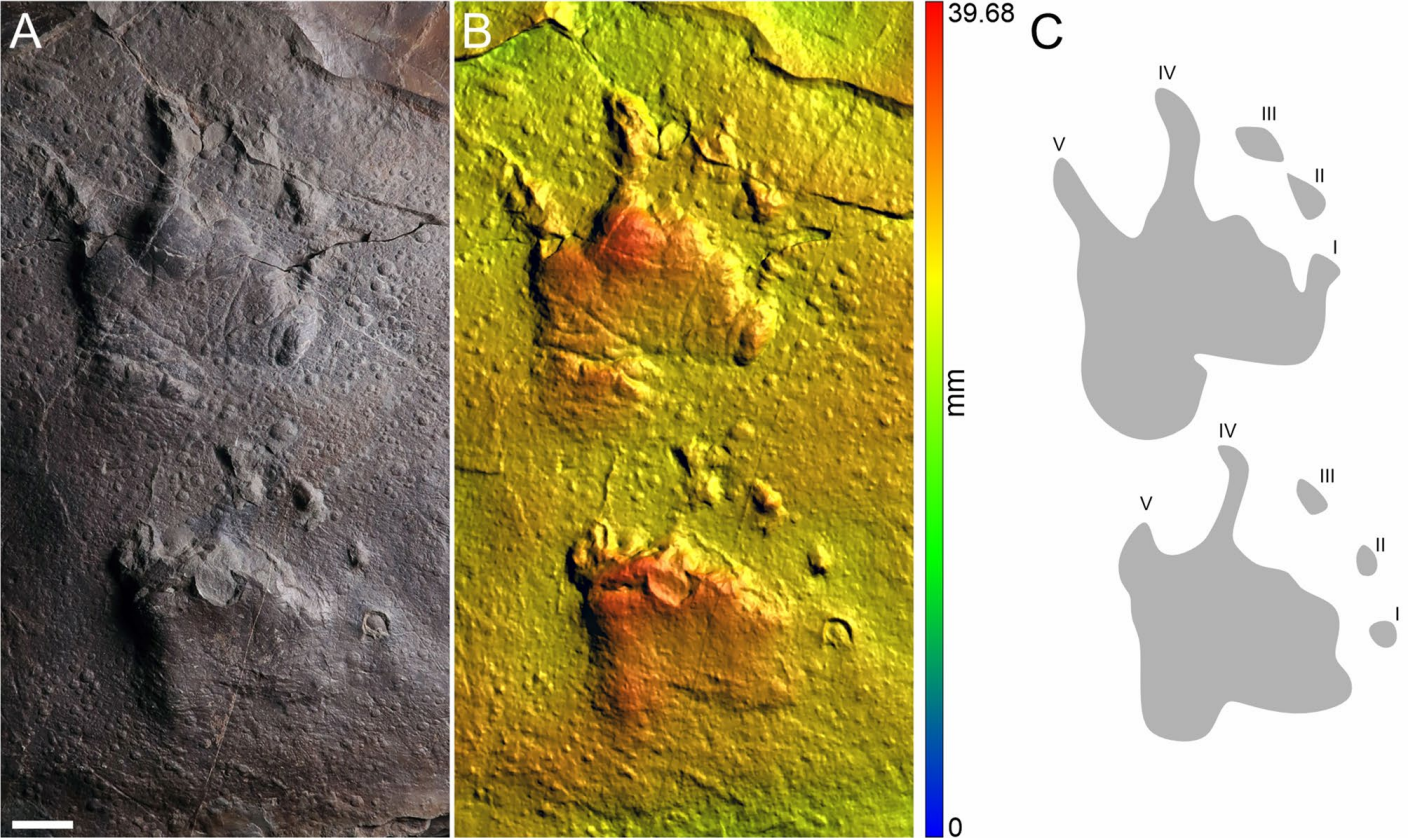
The manus-pes couple of MZM Ge 29610 (convex hyporelief) (**A**), image‐based modeling (**B**), and the outline drawing (**C**). Scale bar 2 cm.

**Figure 3:**
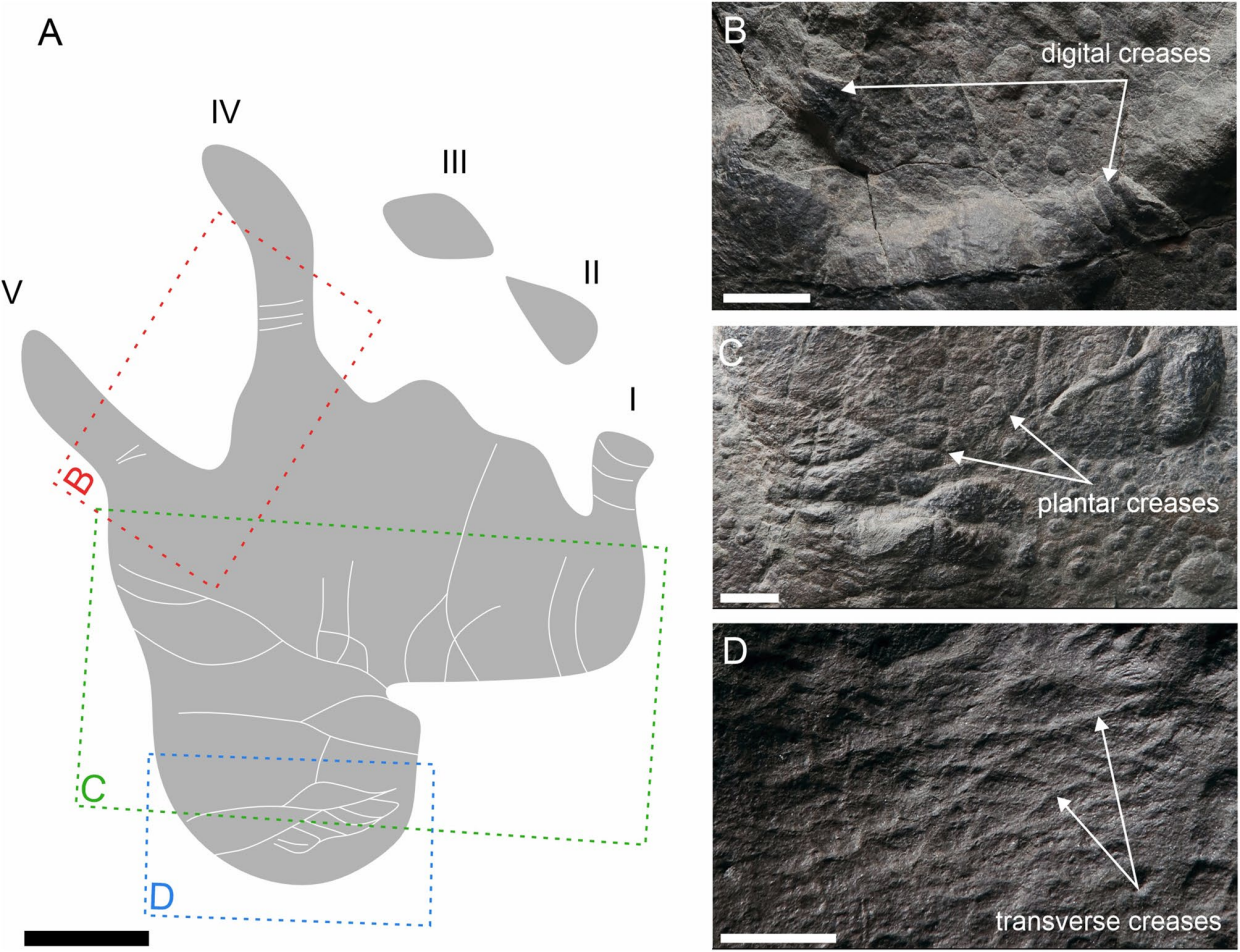
The pes imprint of MZM Ge 29610 with highlighted soft tissue impressions (**A**), including digital (**B**), plantar (**C**), and transverse (**D**) creases. Scale bars: (**A**) 2 cm, (**B**–**D**) 1 cm.

**Figure 4:**
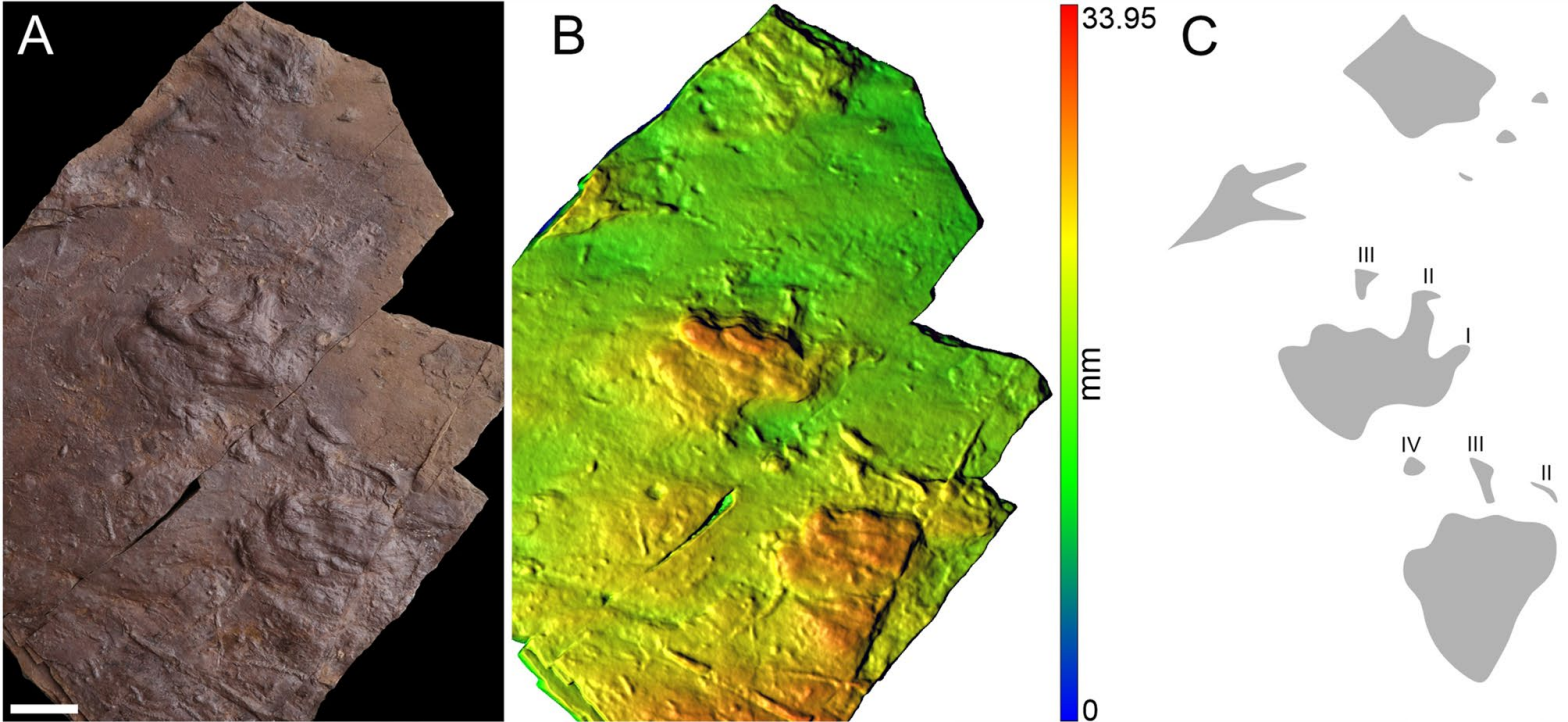
The manus-pes couple and two isolated incomplete tracks of MZM Ge 33072 (convex hyporelief) (**A**), image‐based modeling (**B**), and the outline drawing (**C**). Scale bar 4 cm.

**Figure 5:**
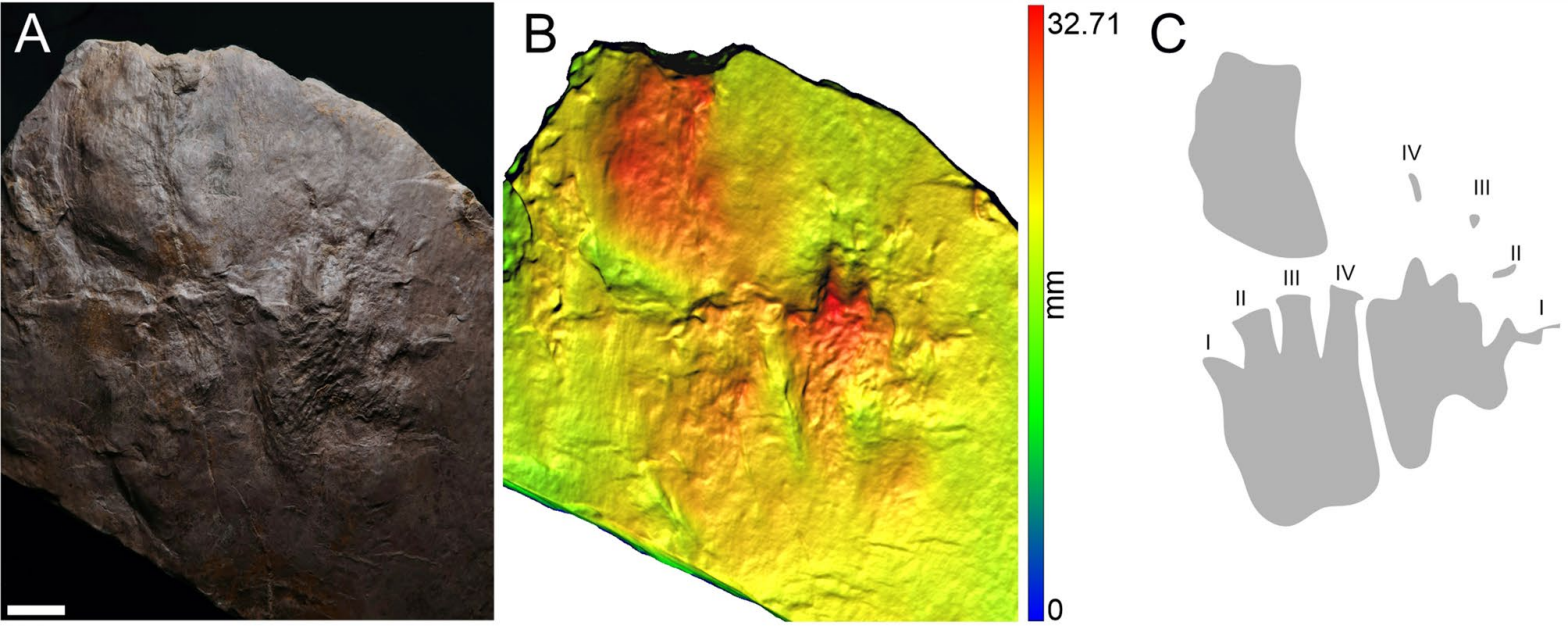
Three isolated tracks of MZM Ge 33073 (convex hyporelief) (**A**), image‐based modeling (**B**), and the outline drawing (**C**). Scale bar 4 cm.

MZM Ge 29610 (Figs. 2, 3), and MZM Ge 33072 (Fig. 4) manus-pes couples, convex hyporelief; MZM Ge 33073 (Fig. 5), isolated footprints, convex hyporelief.

### Occurrence

The specimen MZM Ge 29610 (Figs. 2, 3) originates from a fine grey mudstone of the Lubě Horizon within the lower section of the Letovice Formation (Fig. 1). The specimens MZM Ge 33072 (Fig. 4) and MZM Ge 33073 (Fig. 5) were unearthed from purple-brown, fine-grained sandstones of the upper section of the Padochov Formation (Fig. 1). All specimens are housed at the collections of the Moravian Museum in Brno (MZM).

### Description

Pentadactyl and plantigrade footprints. The manus imprints are as long as they are wide, whereas the pes imprints are longer than wide (Table 1). The pes imprints are about one-fifth longer than the manus imprints. The palm and sole impressions are proximo-distally elongated. The metapodial-phalangeal pads are semispherical and deeply impressed, especially in metacarpals II–V. The digits are relatively short, straight, and with deeply impressed and clawed terminations. Digit I to IV imprints show a continual increase in length. In the best preserved specimen (MZM Ge 29610), the pedal digit V is as long as digit III, whereas the manual digit V is shorter than digit II. Also, the pedal digit V impression of MZM Ge 29610 shows a considerable angle of divergence (Fig. 2). The manus and pes imprints are oriented slightly inward or parallel with the trackway midline. The manus and pes imprints show rather low heteropody. The footprints show median-lateral functional prevalence (i.e., most deeply impressed median-lateral area within the tracks) (Figs. 2B, 4B, 5B). The specimen MZM Ge 29610 shows an inverse arrangement of manus-pes couple; the manus imprint lies behind the pes imprint. The pes imprint of MZM Ge 29610 shows skin impressions, including digital and plantar flexion creases and other transverse creases in the proximal part of the pes impression (Figs. 2, 3).

### Remarks

**Figure 6:**
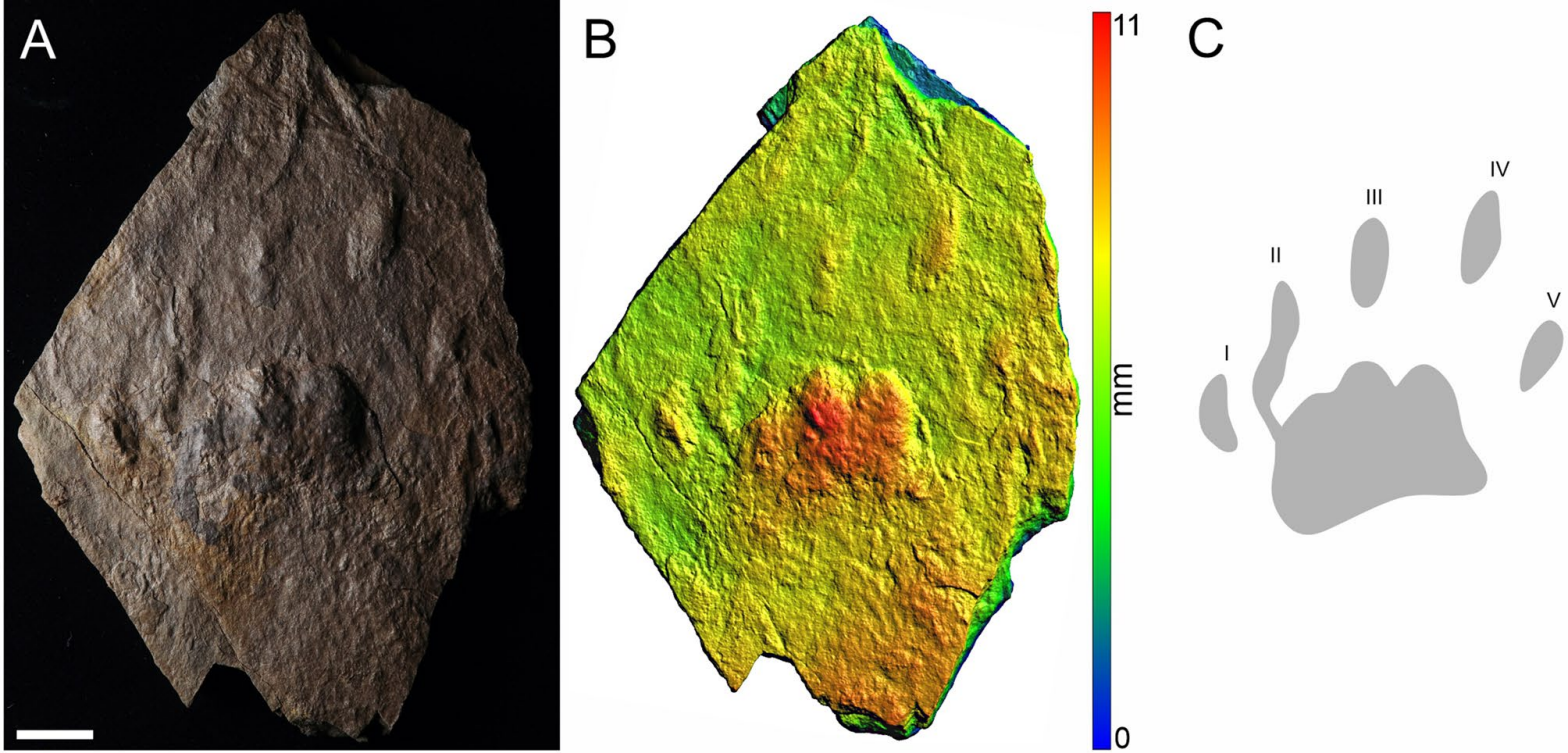
The track of MZM Ge 33071 convex hyporelief (**A**), image‐based modeling (**B**), and the outline drawing (**C**). Scale bar 2 cm.

The best preserved manus-pes couple MZM Ge 29610 was figured by Voigt and Lucas^59^ as a typical representative of *Dimetropus leisnerianus*, although the specimen has never been described in detail. The inverse pattern of the manus-pes couple, observed in this specimen, has previously been described in some couples of the earliest occurring *Dimetropus* isp. trackway from the upper Carboniferous Bochum Formation, Ruhr area, Germany (Ref.^60^, Figs. 5, 6), and is also present in a *Dimetropus* isp. trackway from the Carboniferous of the Cumberland Group, Nova Scotia, Canada (Ref.^61^, Fig. 5 a,b). We assume that the pattern of the manus-pes couple of MZM Ge 29610 is inverse, which is supported by the following features: the pes imprint (which is situated in front of the manus) is significantly larger than the manus imprint; the proximal portion of the sole impression is significantly longer than the palm impression and the impression of the pedal digit V is as long as digit III which is typical for the pes imprint^59^. The manus imprint is also slightly inward-oriented compared to the pes imprint. The imprints of both couples are well-preserved, with deeply impressed proximal as well distal portions. Therefore, we do not expect that they have been distorted taphonomically.

**Figure 7:**
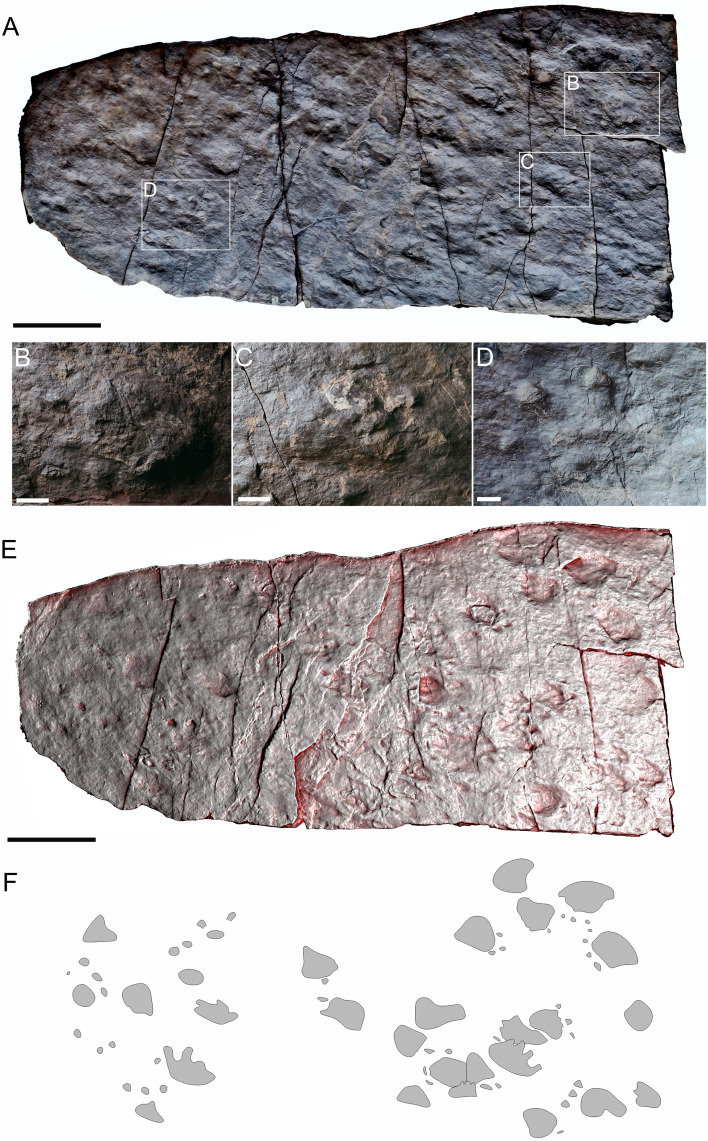
The slab showing (**A**) many incomplete step cycles of MZM Ge 33074 (concave epirelief); (**B**,**C**) *Dimetropus* isp. tracks (convex hyporelief); (**D**) indeterminate track resembling *Ichniotherium* (concave epirelief); and (**E**) image‐based modeling and (**F**) outline drawing of the whole slab. Scale bars: (**A**,**E**,**F**) 20 cm, (**B**–**D**) 2 cm.

The inverted step cycle recorded in MZM Ge 29610 and Carboniferous *Dimetropus* isp. specimens^60,61^ could reflect different body proportions, posture, and locomotion of the trackmakers which would therefore be distinct from those that created non-inverse alternating arrangements of coupled manus-pes imprints, more commonly observed in *Dimetropus* tracks^62–67^. The inverted step cycle was described, for example, in a diadectomorph trackway attributed to *Ichniotherium praesidentis*, and was associated with a longer trunk and a more pronounced sprawling posture of basal diadectomorphs. These contrasted with those of more derived representatives of the clade, such as *Orobates* and *Diadectes*, which have been connected to to *I. sphaerodactylum* tracks (in the case of the former taxon) and *I. cottae* (in the case of the latter taxon), which show non-inverse alternating arrangements (see Ref.^68^).

Skin impressions, preserved in the proximal part of the footprint of MZM Ge 29610, have never been described in early-diverging synapsid footprints; only digital flexion creases are known (see Ref.^62^, Fig. 34a). It is worth noting that other lower Permian material from the Lubě Horizon has been discovered with well-preserved soft part impressions as well; this material has been attributed to the seymouriamorph ichnotaxon *Amphisauropus* and shows digital, palmar, and plantar flexion creases (see Ref.^5^).

The specimens MZM Ge 29610, MZM Ge 33072, and MZM Ge 33073 with their anatomically controlled proportions, with the manus and the pes imprints showing proximodistally elongated heel impressions, semispherical and deep impressions of the metapodial-phalangeal pads, increasing lengths of digits (from I to IV) with deeply impressed clawed terminations, and median-lateral functional prevalence enable the assignment to the ichnotaxon *Dimetropus leisnerianus*.

*Dimetropus* isp.

#### Referred material

MZM Ge 33071 (Fig. 6) isolated footprints, convex hyporelief; MZM Ge 33074 (Fig. 7), incomplete step cycles composed of approximately 20 tracks of *Dimetropus* isp. in at least six different directions (right area of the slab), and 8 tracks of uncertain determination in one direction (left area of the slab), concave epirelief (Fig. 7A,D,E), convex hyporelief (Fig. 7B,C).

#### Occurrence

The specimen MZM Ge 33071 (Fig. 6) derives from a fine brownish mudstone of the Zboněk-Svitávka Horizon of the lower section of the Letovice Formation. MZM Ge 33074 (Fig. 7) was unearthed from purple-brown, fine-grained sandstones of the upper section of the Padochov Formation (Fig. 1). Both specimens are housed at the collections of the Moravian Museum in Brno (MZM).

#### Description

Pentadactyl and plantigrade footprints. The pes imprints are larger than the manus imprints (MZM Ge 33074). The palm and sole impressions are proximo-distally elongated in the specimen MZM Ge 33074 (Fig. 7), whereas in the specimen MZM Ge 33071 is preserved only anterior part of the track (Fig. 6). The metapodial-phalangeal pads are semispherical and deeply impressed. The digit imprints are relatively short, straight, especially preserved as a clawed termination. Digit I to IV imprints show a continual increase in length in MZM Ge 33071 (Fig. 6), whereas some of the footprints within MZM Ge 33074 have digits of similar size (Fig. 7B). The footprints show a rather median-lateral functional prevalence (Fig. 6B, 7E, S1).

### Remarks

The incomplete step cycles of MZM Ge 33074 show features typical for *D. leisnerianus* as well; nevertheless, the tracks are insufficiently preserved to determine the ichnotaxon and some of them are further overstepped. For this reason, we suggest to assign them cautiously to *Dimetropus* isp. The left part of the slab comprising the specimens of MZM Ge 33074 additionally includes a poorly preserved footprints of uncertain determination which show rather long digit impressions with deeply impressed rounded terminations and poorly defined oval palm/sole impressions resembling those of the ichnotaxon *Ichniotherium*. However, owing to the insufficient preservation of this track it is impossible to provide its precise identification at present.

With respect to MZM Ge 33071, its shallow footprints are reminiscent of *Dimetropus* specimens described from the Tambach Formation in Germany (e.g. Ref.^62^, Figs. 36; Ref.^63^, Fig. 7a). Still, due to their poor preservation we prefer to refer MZM Ge 33071 to *Dimetropus* isp. Among the currently known lower Permian ichnotaxa, only *Ichniotherium* and *Limnopus* can reach sizes comparable to those of *Dimetropus* (see Ref.^59^)*.* The manus of *Limnopus*, however, has only four short digits, which are usually well impressed and not separated from their very short palm impressions, while the pes imprint shows a distinctly longer digit III and IV. In turn, the ichnotaxon *Ichniotherium* is characterized through its rather long digits with deeply impressed rounded terminations, and the manual digits II–IV are often bent inwardly. Its deeply impressed elliptical sole and palm impressions and medial-median functional prevalence (e.g. Ref.^69^) also do not match our specimens attributed to *Dimetropus*.

## Discussion

### Taxonomic affinities of the trackmakers

Fossil tracks and trackways attributed to the ichnotaxon *Dimetropus* have been described from numerous strata encompassing the upper Carboniferous and the lower Permian of the United States^63,64,70,71^, Canada^65^, United Kingdom^72^, Germany^60,62,73^, France^66,74^, Poland^75,76^, Italy^77^, Spain^78–80^, and Morocco^81–83^. *Dimetropus* tracks have been generally affiliated with representatives of various non-therapsid synapsid groups, such as early sphenacodontians, ophiacodotids, edaphosaurids, caseids, and varanopids^57,60,62,64,67,73,75,76,84–87^. Even though the phylogenetic placement of Varanopidae among amniotes has been contentious (see Refs.^88,89^), their rather distinct, prolonged digit IV^57,90^ links them with the ichnotaxon *Tambachichnium* (e.g. Refs.^62,63^).

Owing to the high morphological variability of the *Dimetropus* tracks, several ichnospecies have been established in the past, including *D. bereae*^64^, *D. salopensis*^91^, and *D. nicolasi*^74^. All these ichnotaxa are currently considered by some authors to be synonyms of *D. leisnerianus*^62^.

The *Dimetropus leisnerianus* footprints are most commonly considered to belong to sphenacodontids, or early-diverging sphenacodontians in general, which is associated especially with their typically elongated ulnare, a character that is reflected by proximodistally elongated palm impressions of *D. leisnerianus*. In ophiacodontids, caseids, varanopids, and edaphosaurids, the ulnare is typically relatively shorter (e.g. Refs.^57,62,64,73,84–87,92^).

A second, less common *Dimetropus* ichnospecies, *Dimetropus osageorum*^67^ from the Kungurian (lower Permian) of Oklahoma, USA, differs from *D. leisnerianus* in that it shows a high degree of heteropody, short subcircular manus imprint separated into two portions, short, often well-impressed digit impressions which are uniform in length, and rather median-medial functional prevalence in the anterior part of the tracks (see below). The pes imprint is characterized by a single subelliptical to subcircular pad impression in the proximal central part of the sole. Additionally, it has a proximodistally-extended sole impression with deep ‘embayment’ which is also typical, however, for most of the *D. leisnerianus* tracks (see Ref.^62^, Figs. 33). Sacchi et al.^67^ proposed that these features are indicative of a shorter ulnare and a reduced phalangeal formula, which may associate *D. osageorum* with caseids or possibly edaphosaurids, though the latter option is less likely. However, the majority of the ‘smaller-bodied’ European caseids have comparatively longer digits^93,94^, in contrast to ‘larger-bodied’, *Cotylorhynchus*-like forms, such as *Euromycter rutenus* from the Artinskian (lower Permian) of France^95^. Matamales-Andreu et al.^79^ assessed synapsid tracks from the lower Permian of Mallorca, referred to as cf. *Dimetropus* isp., which were also attributed to caseid synapsids, similar to *Ennatosaurus*. In contrast to *D. osageorum*, the cf. *Dimetropus* isp. tracks from Mallorca show longer and slender digits, short sole impressions and a strong outward rotation of the pes imprints^79^. Nevertheless, both these taxa, cf. *Dimetropus* isp. as well as *D. osageorum*, share similar depth pattern, where the functional prevalence was lateral (cf. *Dimetropus* isp.) or median-lateral (*D. osageorum*) in touch-down phases (posterior area of the tracks), whereas in kick-off phases the functional prevalence was changed to the medial-median phases (anterior area of the tracks) in both of them.

A variability in the shape and lengths of the manus/pes imprints and the lengths of digits is observable also in the specimens described herein (Figs. 2, 3, 4, 5, 6, 7), though they still show features which enable their assignment to *D. leisnerianus*, such as proximodistally prolonged heel impressions, rather low heteropody, increase in the length of digits I–IV, and slightly imprinted proximal part of digits. Some of the tracks settled in the incomplete trackways of MZM Ge 33074 show shorter digit impressions; still, no other features diagnostic for the ichnospecies *D. osageorum*, such as separated palm impressions in two portions and the strong heteropody have been observed (Fig. 7A, B). Nevertheless, the relatively short digit impressions were also observed in previously described footprints of *D. leisnerianus* (e.g. Ref.^75^, Fig. 5a; Ref.^78^, Fig. 10; Ref.^96^, Fig. 5b–d).

**Figure 8:**
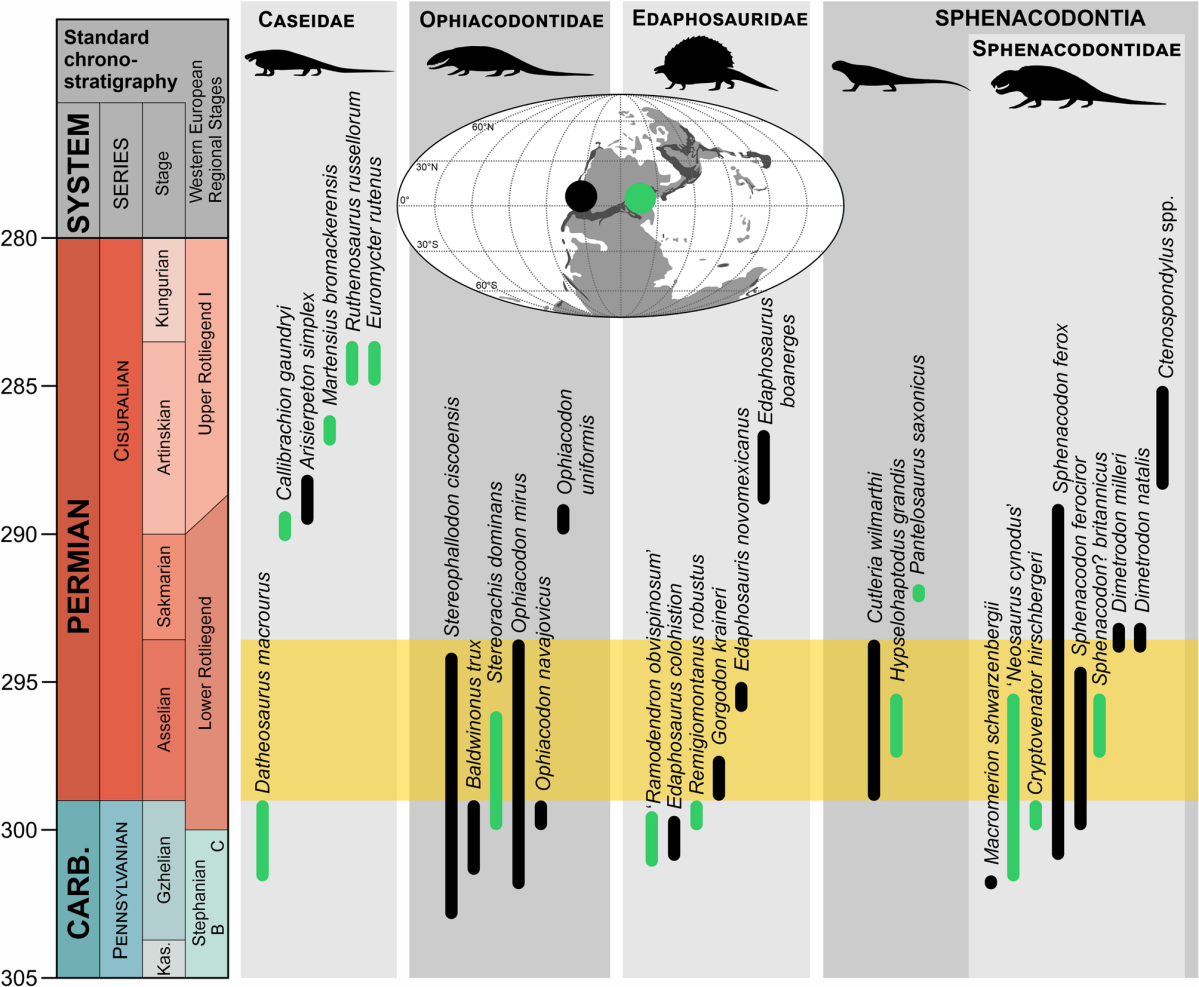
Stratigraphic distribution of medium-to-large-sized early-diverging synapsid species from Europe (green) and North America (black) in the uppermost Pennsylvanian/lower Permian. The time span of the Boskovice Basin is highlighted by orange stripe. The stratigraphic ranges of particular taxa and locality ages are based on Refs.^25,26,99,101–103,109,110^.

Except for the large number of potential trackmakers, the preservation is also affected by many external factors, including the post-registration erosion, character of the substrate (e.g., inclination, water saturation) as well as the trackmaker’s behavior (see Refs.^61,97,98^). Therefore, it is difficult to identify the specific *Dimetropus* trackmakers without considering the body fossils that accompany them in the studied deposits. In the case of the Boskovice Basin tracks and trackways, this is obviously impossible as no early-diverging synapsids have been discovered in the strata to date. The size of the manus-pes couple of MZM Ge 33072 is similar to *Dimetropus leisnerianus* tracks from the Intrasudetic Basin in Poland described by Voigt et al.^75^. We estimate that these tracks were created by an animal that reached the body length (size between the pelvic girdle and the scapular girdle) of about 350 mm. Since the tracks of MZM Ge 29610 and MZM Ge 33074 are larger than MZM Ge 33072 (Table 1), the bodies of their trackmakers must have reached lengths of at least 400 mm. This size corresponds well with most of the medium- to large-sized synapsids of the uppermost Carboniferous–lowermost Permian (Fig. 8).

The members of non-therapsid Sphenacodontia, with their characteristic proximodistally-elongated ulnare, reached the highest diversity during the wetter phases of the early Permian and mainly inhabited equatorial lowland basins^99–101^. While their occurrence in the Boskovice Basin would not be unexpected, given the semi-humid intervals recorded in several horizons (Fig. 1) and a variety of aquatic and terrestrial tetrapods^1,4,13,16,17^, which would represent the potential source of prey, no body fossils of sphenacodontians have been recorded from these strata to date. The only early-diverging sphenacodontian from the Czech Republic is the late Carboniferous *Macromerion schwarzenbergii*^18^ from the Gzhelian of the Slaný Formation in the Kladno-Rakovník Basin.

A complete edaphosaurid autopodium has not been described yet; however, the isolated phalanges of *Edaphosaurus* indicate that the foot was rather broad and short, with strong claws^57^. Edaphosaurids are best known from coal deposits of the Pennsylvanian and their diversity decreased gradually with the continual aridification during the early Permian^26^. The only non-therapsid synapsid body fossil discovered in the Boskovice Basin belongs to the edaphosaurid ‘*Ramodendron obvispinosum*’ from the Stephanian C^22^. The last European occurrence of Edaphosauridae originates from the Gzhelian–Asselian boundary^26^. Therefore, their presence in the Asselian of the Boskovice Basin is less likely.

The members of Caseidae are known in Europe since the uppermost Carboniferous; still, most of their specimens originate from the upper part of the lower Permian^95,102,103^. Caseids belong to the longest-survived non-therapsid synapsids, and were more resistant to the increasing aridification in the middle Permian^104–106^. The nearest occurrence of caseid synapsids is represented by *Datheosaurus macrourus*, which derives from the Gzhelian of the Intrasudetic Basin, Poland^94^.

Ophiacodontids are extremely rare in Europe. They are represented by the upper Carboniferous taxon *Archaeothyris*, which was found in the Pilsen Basin, Czech Republic^24^, the upper Carboniferous ‘*Stereorachis*’ *blanziacensis* (Blanzy-Montceau Basin^107^), and the lower Permian *Stereorachis dominans* (Igornay near Autun, France^108^).

Owing to the spatiotemporal distribution of non-therapsid synapsids (Fig. 8) and the apparent morphological disparity of the *Dimetropus* footprints from the Boskovice Basin, it is impossible to assign the studied tracks beyond early-diverging, or ‘pelycosaur-grade’, Synapsida at present.

## Conclusions

The Asselian (lowermost Permian) strata of the Boskovice Basin in the Czech Republic have long been renowned for their extraordinarily rich fossil record of aquatic discosauriscids, some of which with exceptional preservation of soft tissues. Discoveries of other tetrapods have been very rare and are limited to a few temnospondyls of rather contentious taxonomic affinities.

However, recent fieldwork in the Asselian deposits of the Letovice and Padochov formations have yielded numerous assemblages of tracks and trackways, highlighting previously unrecognized diversity of the Boskovice Basin tetrapods. Here, we describe tracks and trackways clearly pertaining to early-diverging, or ‘pelycosaur-grade’, synapsids, thus securing the first evidence of mammal-line amniotes in the Permian of the Czech Republic. All of the studied specimens can be attributed to the ichnotaxon *Dimetropus* and, with an exception of two specimens, all show characters diagnostic for *Dimetropus leisnerianus*. Furthermore, the best-preserved specimen MZM Ge 29610 shows skin impressions, such as plantar flexion creases and other transverse creases, which have never been described in *Dimetropus* tracks before.

The footprints attributed to *D. leisnerianus* are most commonly treated to represent those of early-diverging sphenacodontians, and such affinities may indeed be plausible for the Boskovice Basin specimens as well. However, owing to the absence of synapsid body fossils in the track-bearing strata, and overall morphological variability of *D. leisnerianus* tracks, we refrain from identifying the tracks beyond non-therapsid Synapsida.

Regardless, the abundance, preservation, and taxonomic affinities of the studied material stress the significance of fossil tracks and trackways discovered in the Asselian of the Boskovice Basin for reconstructions of tetrapod faunas recorded in paleoequatorial basinal deposits.

## Data Availability

All data generated or analyzed during this study are included in this published article. Three-dimensional models have been uploaded to MorphoSource and are accessible through the following link: https://www.morphosource.org/projects/000489812/about?locale=en.
